# Quest Narratives and Heroine Journeys: the road to freebirth and the joy of undisturbed physiological birth

**DOI:** 10.3389/fgwh.2024.1495914

**Published:** 2025-01-30

**Authors:** Gemma McKenzie

**Affiliations:** Florence Nightingale Faculty of Nursing, Midwifery and Palliative Care, King’s College London, Waterloo Campus, London, United Kingdom

**Keywords:** freebirth, undisturbed physiological birth, voice-centred relational method, VCRM, narrative research, I-poetry, Quest Narratives, Heroine Journey

## Abstract

Freebirth (intentionally giving birth without doctors or midwives present) is a taboo and stigmatised birthing decision. In this study, 16 women who had freebirthed their babies in the UK were interviewed, and the data was analysed using the voice-centred relational method (VCRM). This methodology produces a range of outputs, including I-Poetry. Previous scholars have noted the difficulty in presenting results from VCRM research and have devised varied and creative ways of subverting these obstacles. Uniquely, this article relies on the theories of Joseph Campbell, Arthur Frank, and Kim Hudson to present women's freebirth accounts as both Quest Narratives and Heroine Journeys. The article outlines the theoretical underpinnings of the Heroine’s Journey and demonstrates its use with women's freebirth accounts as they pertain to the joy of undisturbed physiological birth.

## 1 Introduction

In Kate Chopin's novel “The Awakening”, first published in 1899, she describes the final summers in the life of her protagonist Edna Pontellier. Edna is a creative woman suffocated by the social expectations inflicted on her by nineteenth-century New Orleans society. The story recounts Edna's rebellion against gender expectations and her struggle to live autonomously whilst limited by patriarchal norms [see Khan (1)]. Recognised as early feminist literature [see, for example, Streater (2)], the novel depicts a now well-recognised narrative arc that frequently appears in Western storytelling, labelled the Heroine's Journey [for further examples, see Hudson (3)].

A similar struggle is evident in women's attempts to freebirth. Freebirth occurs when women intentionally give birth without doctors or midwives present in societies where there are maternity services available to assist them (4). However, the taboo nature of the act [see, for example, Holten and De Miranda (5)] means that women must overcome social disapproval and other obstacles to give birth in this way.

I carried out narrative interviews with 16 women who had freebirthed their babies in the UK. Analysed using the voice-centred relational method (VCRM) (6), previous scholars have recognised the limitations inherent in presenting data using the methodology (7). As a way of overcoming these problems, I draw from the work of Campbell (8), Frank (9), and Hudson (3), combining ideas related to the Quest Narrative and the Heroine's Journey to present results from The Freebirth Study as they pertain to the joy of undisturbed physiological birth.

The aim of this article is, therefore, to demonstrate the use of the Heroine's Journey as a pathway to both heal the shortfalls of the VCRM and to present results. Although the article takes the format of a typical social science paper, much emphasis is placed on the explanation of the Heroine's Journey and the methodology. In addition to existing freebirth literature, the data is largely contextualised against the methodological theory. This is to introduce the reader to an original way of presenting qualitative data and particularly that generated using the VCRM.

This article consists of three parts. The first explains the basic VCRM methodology, the way in which the data was captured and analysed, and introduces the limitations in the presentation of the VCRM findings. The second part explains the Heroine's Journey and its underlying theory. The third part then presents the findings of The Freebirth Study as they pertain to the joy of undisturbed physiological birth using the Heroine's Journey as the underpinning framework.

## 2 Aims of the study

The aim of The Freebirth Study was to understand women's experiences of freebirthing in the UK. This paper presents findings related to the joys of physiological birth, in addition to an innovative methodological approach.

## 3 Ethics

Ethical approval for the study was granted by the King's College London PNM Ethics Committee on 8 October 2019, number HR-19/20-13511.

### 3.1 Part 1: methods

Following the co-creation of a recruitment poster with a service user and its advertisement on Facebook, I conducted 16 narrative interviews with women who had freebirthed their babies in the UK. Interviews were carried out at the participants’ homes, their workplaces, and, on one occasion, at a community centre. As the interviews were narrative in nature, the women were asked to simply tell their freebirth stories. The women could, therefore, start where they felt appropriate and provide whatever information they felt relevant. Interviews were transcribed, anonymised, and pseudonymised, with all interviewees choosing their own pseudonyms.

## 4 The voice-centred relational method

In 1982, Carol Gilligan wrote a book entitled *In a Different Voice* (10)*.* In it, she applied both literary theory and psychology to understand “the relation between judgment and action in a situation of moral conflict and choice” (p. 1). Her interest was in women's voices, how they are silenced, and how they silence themselves. She describes a “male-voiced civilization” (p. xi) in which women are fearful of being condemned, hurt, or misunderstood if they voice their desires or thoughts. In this vein, according to Gilligan (10), women perceive that it is better to remain silent to “keep the peace” (p. x).

Expanding on these ideas, Brown and Gilligan (6) developed the VCRM. Their aim was to understand women's psychological development by exploring girls’ experiences during childhood and adolescence. More specifically, they wanted to understand the role of relationships, particularly with regard to how women silence themselves to avoid open conflict, disagreement, isolation, or violence (p. 3). In their view, this silence results in a loss of voice “for the sake of becoming a good woman” (p. 2).

From a practical perspective, the VCRM is a qualitative, narrative methodology that differs considerably from a general thematic analysis. In essence, the researcher undertakes four readings—also called listenings (11)—of their interview data. These consist of

1. the plot and the researcher's response to it;
2. the “Voice of the I” and I-Poetry;
3. understanding relationships; and
4. the social, political, cultural, and structural context.

### 4.1 The plot and the researcher's response

The plot is of central importance to any form of narrative as it “knit(s) events together,” allowing us to understand the deeper significance of an event in the light of others [(12), p. 170]. Drawing on the literary aspects of the VCRM, there is a need to understand the “basic” [(13), p. 3], “dominant” [(11), p. 160], or “main” [(14), p. 66] themes of a narrative.

Regarding the researcher's response, the purpose of this is to

“… reflect on ourselves as people in the privileged position of interpreting the life events of another and consider the implications of this act.” [(6), p. 27].

Although VCRM researchers would typically use hard copy transcripts and highlight important areas of text using various colour-coded markings (11), in The Freebirth Study, this was impractical, and notes were kept electronically. This included summaries of each participant's story, the characters involved, and my reflections on women's accounts.

### 4.2 The “Voice of the I” and I-Poetry

A detailed description of this reading has been published elsewhere (15). In short, the creation of I-poetry requires the researcher to highlight in the transcript where the interviewee refers to themselves with reference to “I.” The purpose of this is to privilege the participant's position in the narrative. However, there are some limitations to this, and researchers have begun to include “we” (16) and “she” poems (17). Recognising the importance of different pronouns to reflect oneself, in The Freebirth Study, words such as “me,” and “myself” were retained as versions of “I.”

### 4.3 Understanding relationships

There is very little literature attuned to the nuances of the third reading and its output. Authors with a specialism or interest in psychology tend to interpret the third reading as “Listening to Contrapunctal Voices” (13, 14, 18, 19). This requires the researcher to label the different “voices” they hear an interviewee using in their account [(18), p. 79]. In contrast, an alternative and more sociological interpretation of the third reading shifts the emphasis of the analysis from the internal to the external. This is demonstrated by Mauthner and Doucet (20) and can be explained by the description they give of their own “version” of the VCRM. It consists of an analysis that explores

“… individuals’ narrative accounts in terms of their relationships to themselves, their relationships to the people around them, and their relationship to the broader social, structural and cultural contexts within which they live.” (p. 126).

Uncomfortable with labelling women's perceived “voices,” in The Freebirth Study, I approached the data from the latter perspective, specifically questioning in which relationships the women's voices were heard and in which were they silenced. The character lists from the first reading were, therefore, useful, and notes were made on how and with whom the women were able to openly express themselves, their thoughts, feelings, and plans.

### 4.4 The social, political, cultural, and structural context

During the fourth reading and following the approach of Mauthner and Doucet (20), an analysis was made of the “respondents’ accounts and experiences within broader social, political, cultural and structural contexts” [(20), p. 132]. Given the feminist lens through which I was analysing the data, transcripts were contextualised against existing bioethical, legal, and sociological literature. This enabled the women's experiences to be understood against the backdrop of academic thought.

## 5 Reflexivity

Although the second reading of the VCRM allows for an explicit form of reflexivity, in qualitative research, reflexivity should be a “continual internal dialogue” [(21), p. 220]. This was maintained with a reflexivity journal but also via an acknowledgement of my positionality. My own journey to The Freebirth Study was initiated by appalling obstetric care during the birth of my children and my search for answers via my relationship with the national charity Association for Improvements in the Maternity Services (AIMS). AIMS supported the project from its inception and during the course of the study, I volunteered with the charity. This included a 3-month internship funded by the Economic and Social Research Council (ESRC), enabling me to forge relationships with doulas, midwives, activists, mothers, and other birth-related workers. The project also included significant public and patient involvement, including a co-created short film (22) and a graphic zine.

## 6 Presenting the findings from a VCRM study

There are problems inherent in the presentation of a VCRM study. One major issue is the lack of guidance on writing up the findings. Any form of writing needs a thread that links the elements of the narrative together. Within the four readings, however, there is no obvious thread except the concept of “voice.” Even with this, “voice” still needs to be attached to a meaningful anchor around which the data can be both presented and discussed. Whilst the literary roots of the VCRM lend themselves well to narrative approaches that emphasise the importance of storytelling, with no thematic analysis, it is not obvious how to find and present connections between those stories. How a researcher links plot, poetry, an analysis of relationships, and the social context in which they take place remains unclear.

Inevitably, this is reflected in the lack of academic literature containing full VCRM studies. In fact, much of the VCRM literature consists of methodology papers that do not contain an overview of the findings of a study (14, 17, 23–26). Further, papers in which research results are presented are often limited to the use of case studies [see Pinto (27), Paliadelis and Cruickshank (28), Byrne et al. (29), and Shergill (30)]. Some researchers have simply lifted aspects of the VCRM but have not employed the methodology in any full or formal sense. This is most notable in relation to the use of I-Poetry [see Brown (31) and Miller et al. (32)], thus indicating the problematic nature of trying to locate studies from which to draw appropriate presentation formats.

A consequence of these issues is that researchers often resort to creating additional readings or using another approach to ensure their data can be appropriately presented. Smith (7) highlights the difficulty she had creating a workable format and notes this issue as a “significant limitation” to the methodology (p. 152). Nevertheless, some researchers have developed creative ways to overcome obstacles pertaining to VCRM data presentation. Mauthner and Doucet (20), for example, struggled to carry out four readings of all their data and resorted to creating summaries, case studies, and “break(ing) up each transcript into a number of overlapping themes and sub-themes” (p. 135). Montgomery (33) took a similar approach and carried out a fifth reading that incorporated a thematic analysis. Similarly, Shergill (30) used a thematic analysis alongside a detailed case study. Inspired by the work of Frank (9), Smith (7) also carried out a fifth reading, which she called “letting stories breathe” (p. 151). This resulted in her drawing women's stories together by recognising shared narratives within them.

My own novel approach resulted in the use of the Heroine's Journey as a framework. This will be explained and presented below.

### 6.1 Part 2: the Quest Narrative and the Heroine’s Journey

All stories consist of a few common structural elements found universally in myths, fairy tales, dreams, and movies. They are known collectively as the Hero's Journey [(34), p. xiv].

The foundations of the VCRM have a rich theoretical heritage linked to psychology and literary theory which are only infrequently drawn upon in academic texts. This is particularly relevant to the understanding of plot which is based on a huge body of work pertaining to narrative thought.

One narrative approach appeared in 1995 when Arthur W. Frank published the first edition of his noted work *The Wounded Storyteller*. Grounded in both narrative and literary theory and playing an important role in the understanding of healthcare as experienced by patients, Frank (9) framed ill people's stories within three general narratives. These included the Restitution Narrative, the Chaos Narrative, and most relevant to this study, the Quest Narrative. Frank formulated these ideas by creating “a kind of memoir” of his own experiences of testicular cancer and “cultivat[ing] the stories of others who are wounded” (p. xxi). He writes of the relevance of “voice” and emphasises the importance of how “(t)hose who have been objects of others’ reports are now telling their own stories” (p. xxi).

Pausing momentarily—and to avoid undermining decades of feminist scholarship—pregnancy is not an illness. Pregnant women, therefore, cannot be perceived to be inhabiting the same space as the patients in Frank's work. However, an interesting parallel can be drawn between the narratives in this study and both Frank's ideas and the foundational theories underpinning his thesis. Frank based his ideas regarding Quest Narratives on Joseph Campbell's hugely influential classic 1949 text *The Hero with a Thousand Faces*. Campbell (8) argued that mythological narratives share a general structure, which he described as the “monomyth” (p. 1). In brief, the hero of the monomyth undertakes a journey into unfamiliar territory. He may encounter a protective figure who assists him in navigating the terrain and undergoes a succession of trials. Overcoming these and ultimately transformed, the hero receives the “ultimate boon” (p. 148) in the form of knowledge that he must share with others. On returning home, the hero is transformed by his experience and the “master of the two worlds” (p. 196). Obvious examples of contemporary plots depicting the structure of such a hero's journey include *Star Wars* and *Lord of the Rings* [for further examples, see Rubin (35) and Morong (36)].

Frank (9) applies this structure to his concept of the Quest Narrative. While many of the more detailed aspects of Campbell's (8) work are inapplicable, for example, his writing on the hero's “atonement with the father” (p. 105), Frank (9) draws directly from other aspects such as “the road of trials” (p. 81). For Frank (9), this is the various “sufferings that illness involves” (p. 118). What Frank has, therefore, successfully done is take the spirit of *The Hero with a Thousand Faces* and evolve it into a framework that he has applied to illness narratives.

## 7 The theories of Carl Jung

Before taking the thread of Campbell's (8) work any further, it is important to briefly outline its theoretical foundations. The work of Carl Jung underpins both the work of Campbell and many of the later theories that stem from Campbell's ideas, particularly those pertaining to the Hero’s and Heroine’s Journey (see below regarding the latter). Central to Campbell's (8) monomyth is Jung's theory of the “collective unconscious” [(37), p. 152]. This “consist[s] of mythological motifs or primordial images, for which reason the myths of all nations are its real exponents” (p. 152). According to Jung, therefore, the collective unconscious is part of the human psyche which reflects the accumulated experiences of the whole of mankind; it thus transcends culture and individual experiences. The proof of the collective unconscious manifests in what Jung describes as “archetypes” (38), and these appear symbolically and mythically in all cultures throughout the world, both past and present.

Jung (37) recognised a range of archetypal figures, including “the great mother, father, child, devil, god, wise old man, wise old woman, the witch, the trickster, the hero” [(39), p. 137]. Whilst Jung's ideas have been critiqued [see, for example, Neher (40)], they have had an impact within the world of psychotherapy and within the realms of narrative, storytelling, and literary theory. In essence, scholars have explored how these universal archetypes consistently appear in myth, legend, fairy tales, and contemporary literature and film [see, for example, Merritt et al. (41), Obidič (42), Gaarden (43). and Orenstein (44)]. Further, overlapping with psychological thought is the argument that the appearance of these archetypes continues to have relevance to the way we live and understand our own lives today (45, 46). The point, therefore, is that Jungian theory emphasises the ubiquitous nature of archetypes such as the hero. In turn, this provides the foundation for universally identifiable and meaningful human stories such as the monomyth recognised by Campbell (8).

## 8 The Heroine’s Journey

Returning to Campbell's (8) theory, it is questionable as to whether he understood the hero's journey to be applicable to both men and women (47) although he does provide female examples in his argument [see, for example, his discussion on the Mesopotamian goddess Inanna (8), p. 87–90]. Nevertheless, some authors have noted the androcentric nature of Campbell's conclusions (2, 48–50). In response, alternative Heroine Journeys have been suggested (30, 47, 48, 51, 52). Whilst all feminist in nature, not all are academic texts and they vary in discipline, ranging from, for example, eco-feminism to literature and screenwriting. However, mirroring Jungian theory, what links these texts is the authors’ view that the Heroine’s Journey repeatedly appears within storytelling and/or can be used as symbolic of women's real-life struggles. As Carriger (51) notes, the heroine's journey “is *endemic* to Western culture” (p. 5) (italics in original).

One notable example appears in the 2010 text *The Virgin's Promise* by the narrative theorist Kim Hudson. Similar to Campbell's work, Hudson (3) based her ideas on Jungian archetypes and argues that her concept is applicable to both male and female characters. Her reference to the heroine is, therefore, thematic as opposed to gender specific. What distinguishes Hudson's virgin from Campbell's hero is that the “feminine” journey is internal, while the “masculine” one is external. As Hudson (3) writes

“The Virgin must answer the question: Who do I know myself to be and what do I want to do in the world, separate from what everyone else wants of me?” (p. 7).

Crucially, in Hudson's (5) work, the Virgin “must stop conforming to the wishes or beliefs of others or suffer greatly” (p. 21), and her “challenge is to face the influences of her domestic world” (p. 23). Hudson (5) describes the problem the Virgin confronts

“The obstacle in the Virgin story is the people around her who want to control her actions. The Virgin is not a volunteer in this adventure; rather, the plan for her life is the central theme. No one is encouraging her to take action: in fact, they are strongly discouraging it. While the kingdom wants her to be passive, the Virgin wants to actively pursue her own path.” (p. 24).

According to Hudson's (5) theory, as the Virgin moves forward in her journey, she realises that the price of conformity is the suppression of her true self. She has an “Opportunity to Shine” which compels her into action (p. 42). “Dressing the Part,” (p. 46) the Virgin has the chance to sample what it feels like to be true to herself. She creates a “Secret World” “separate from external authority” (p. 50) and where she has a frequent “fear of discovery” (p. 53). Circumstances somehow change, and this secret world is exposed, whereby the Virgin experiences psychological growth. Ripple effects due to this change create chaos and the Virgin experiences doubt before deciding to trust herself. Her value is then recognised and “The Kingdom is Brighter” (p. 75).

While Hudson (3) created her theory for the benefit of screenwriters, the Jungian foundations of the concept mean that archetypes such as the Virgin “model pathways for the universal transformations in life” (p. 9). Drawing parallels with fairy tales, Hudson (5) cites Bettelheim (53), arguing that both these and her own Heroine's Journey reflect “stories of casual, everyday life events, which take place in the domestic realm” (p. 7). Hudson (5) uses an example from her own life to demonstrate the theory (p. xvii) but also numerous Hollywood examples to prove her point. These include, for example, *Bend It Like Beckham* (p. 80), *Brokeback Mountain* (p. 83), *Legally Blonde* (p. 87)*, Pretty Woman* (p. 91), and *Sister Act* (p. 93).

Given the literary foundations of the VCRM, examples that may better suit this article are Margaret Atwood's (54) *The Handmaid's Tale* (55), *The Awakening*, Silvia Plath's (56) *The Bell Jar,* or Charlotte Perkins Gilman’s (57) *The Yellow Wallpaper*. These works have been analysed by numerous scholars who recognised the female protagonists as struggling to retain their identity in an oppressive and suffocating society, whose norms expect them to behave in ways contrary to their own true natures. Notions of power, panopticism, patriarchy, and gendered oppression appear in these analyses as obstacles holding back the protagonists [see, for example, Bak (58), Stilman and Johnson (59), Cooper (60), Oakley (61), and Clark (62)]. Chopin describes her protagonist Edna Pontellier in *The Awakening*, in nineteenth-century New Orleans, as living a “dual life—that outward existence which conforms, the inward life that questions” (p. 13).

Although Hudson (3) clearly delineates between the Hero’s and the Heroine's Journeys, not all authors do. Vogler (34) considers Joseph Campbell's work to cover both “inward” and “outward” journeys (p. 7). In other words, from his perspective, the hero refers to any gender and can incorporate a journey of “the mind, the heart, the spirit” (p. 7). Regardless of which label one applies to the narrative template, the point remains: successful storytelling features elements of a general formula that appears throughout time and culture, and this can be used to provide meaning regarding the complexities of human life.

Neither Campbell (8) nor Hudson (5) required their template to be prescriptive. Even Frank's (9) Quest Narrative only reflects the spirit of Campbell's work. As Vogler (34) notes, “[e]very storyteller bends the mythic pattern to his or her own purpose” (p. 7). With this in mind, I have combined the ideas of both Hudson (5) and Campbell (8) to form a template for the Freebirth Study and its VCRM approach.

As will be demonstrated in the following paragraphs, the women in this cohort recognise the stigmatised nature of their desire to give birth in ways that best reflect their own values. Consequently, there are moments of conformity, resistance, and soul searching. In these stories, the women are often silenced, conflict erupts, obstacles must be overcome, and secret worlds are formed by connecting to a network of similarly minded individuals who share their knowledge and experiences. Although the journey is not easy, ultimately, the women succeed in their goal of undisturbed physiological birth.

### 8.1 Part 3: results and discussion as per the Heroine’s Journey template

This section of the paper presents the results of The Freebirth Study as they pertain to the joys of undisturbed physiological birth. Drawing from the ideas of Campbell (8), Frank (63), and Hudson (5), the template used to present the results includes the following stages:

a. The Dependent World (Hudson).
b. Price of Conformity (Hudson).
c. The Road of Trials (Campbell and Frank).
d. Engagement with the Female Network, including Hudson's Secret Worlds.
e. Apotheosis (Campbell and Frank).

Although the results could have been presented with additional stages, such as The Kingdom is Brighter, for the purposes of this paper, these stages have been included as they are key points in the women's freebirth journeys. Combined, they demonstrate the general point that the women's accounts reflected the Quest Narrative as per the ideas of Frank (9).

(a) The dependent world

The beginning of the Quest Narrative is the Dependent World [(3)], which is the social realm in which the protagonist lives. It can be a force or external authority, in addition to a belief the protagonist holds (p. 30–31). One way in which this force or belief materialises is via social convention. This can include cultural values or practices, traditions, and defined gender roles [(3), p. 32–34]. At the heart of Hudson's (5) Heroine's Journey is the need for the protagonist to “separate themselves from the power structures they were born into” (p. 11).

This sits at the centre of the quest on which the participants were to embark. Even though there is a legal right to freebirth, this does not equate to it being considered socially acceptable. Previous studies have highlighted how freebirthing is considered “deviant” behaviour [(5), p. 60], and women may “face moralistic opposition [and] accusations of irresponsibility” [(63), p. 10]. Notions of how good mothers are expected to behave include the presumption that they are obedient to medical professionals [(64), p. 530] and are self-sacrificing [see, e.g., Wolf (65) and McNolty and Garrett (66)]. As Kukla (67) affirms, during birth, the “good” mother “mak[es] proper, risk-averse, self-sacrificing choices” (p. 74). In other words, she displays a “foetus-first mentality” [Milne (68)]. As such, therefore, the path to freebirth is problematic as social norms may inhibit a woman from expressing her desire and exercising her right to pursue this course of action.

Although this was the social world in which women were operating, this was not immediately apparent to them. No woman started her first pregnancy—and in most cases, her freebirth pregnancy—wanting to freebirth. This was something that emerged as they became more aware of the Dependent World.

Within the cohort, four participants were first-time mums when they freebirthed, and the remaining participants had between two and four children. Table 1 demonstrates the birthing experiences of the cohort.

**Table 1:** Participants' birthing experiences.

Participant	First birth	Second birth	Third birth	Fourth birth
Alicia	Hospital	Hospital	Homebirth	Freebirth
Bianca	Hospital	Freebirth	—	—
Cat	Homebirth	Freebirth	—	—
Danielle	Hospital	Homebirth	Freebirth	—
Elsie	Hospital	Homebirth	Homebirth	Freebirth
Fionnuala	Homebirth	Freebirth	—	—
Georgia	Freebirth	—	—	—
Heather	Homebirth	Homebirth	Freebirth	—
Ivy	Freebirth	—	—	—
Jiskra	Homebirth	Freebirth	—	—
Kitty	Hospital	Freebirth	—	—
Leah	Hospital	Homebirth	Freebirth	—
Marion	Freebirth	—	—	—
Nadia	Hospital	Birthing centre	Birthing centre	Freebirth
Ophelia	Hospital	Homebirth	Homebirth	Freebirth
Polly	Freebirth	—	—	—

As the interviews were narrative in form, the women could begin their stories at whatever point they felt important. Demonstrating their understanding of birth in the UK, some women began their stories with tales of their own births:

***Alternative birth***

I knew then that

I had to, sort of, do some work.

I thought

I know that what happened

I knew that what had happened

To my mum

And my birth wasn't right.

I knew that it didn't have to be like that.

*Georgia*

Other women discussed their expectations of childbirth before they became pregnant. Elsie indicated an intuitive mindset in which she always “felt she knew how to have babies, even though I'd never had them before.” Alicia described how she envisioned her first birth to be

“[M]y dream before I had children was to have a water birth at home, and that was when I was, you know, nineteen. I was dreaming about things like that way before I was married and having a baby. I just dreamed of having this friend, I suppose, just with me, just stroking my hair-, I know it sounds crazy, stroking my hair, low music, low lighting.”

In contrast, Ophelia had not thought about the type of birth she wanted until a few weeks before her first (non-freebirth) child's due date. These accounts begin to weave a tapestry of the previous knowledge and expectations the women had of childbirth and their mindset prior to their first pregnancies. They also highlight the diversity of the women's initial views and indicate the heterogeneity of the interviewees’ starting points. What is important, however, and indicates the power of social convention, is that no participant envisioned freebirthing prior to their first pregnancy.

(b). Price of Conformity

Hudson (5) discusses the second stage in the protagonist's journey as the Price of Conformity (p. 36). This can include agreeing to live within restrictive boundaries (p. 37), living a life of servitude, and facing psychological danger (p. 40). The protagonist may, therefore, conform to expected standards of behaviour and become “out of sorts with her true self” (p. 41). In other words, when she “subscribes to the views of the people around her, she experiences a loss of self” (p. 36). At this stage, the protagonist is learning about what is expected of her and the consequences of following a predetermined pathway.

It was at this juncture in The Freebirth Study that interviewees outlined their previous experiences with maternity care, either during earlier pregnancies or during their freebirth one. It would be incorrect to presume all the participants had experienced poor previous care or obstetric violence. Although that was a feature in many accounts, the women's experiences were more complicated than could be described as simply “good” or “bad.” Their narratives were undulating with both highs and lows, and these often appeared during the same event or with the same healthcare professional (HCP). Even when women had been subjected to the worst forms of obstetric violence, they often also reported a good experience with a caring postnatal midwife or member of the maternity team. This complexity is demonstrated by Jiskra's account of being taken to the hospital for the manual removal of her placenta after her first midwife attended homebirth:

“… when the ambulance arrived, they put me on a wheelchair … and then they wheeled me through a public area. I was mess-, I was a total mess, you know, … I just, I just started wailing, like, weeping, like, loudly, and it was just such a humiliating situation, but, also, like, I just, kind of, really-, I just lost it, totally. And then, err, they took me to the labour ward and, um-, and I was waiting, and they started putting the cannulas in my, in my arms, and I hate, I hate needles, and I'm just really quite squeamish about this, sort of, stuff, and just, again, like, this restrain, being restrained, kind of, wired to things.”

Throughout this frenetic experience of emotion, pain, and resistance, Jiskra was also attended by a midwife she described as

“… very maternal. She was this, kind of, beautiful woman-, beautiful middle-aged woman with a really, kind of-, I just felt, like, kind of, love pouring. I don't know, I mean, that was just the way I, kind of, felt about her.”

To note, the women's previous births were not always abusive or traumatic. For two women, earlier precipitous births were an important factor. During her previous birth, Cat enjoyed a homebirth with two midwives and an “amazing student.” At one of her homebirths and according to her wishes, Elsie laboured in her bedroom while her midwife “just sat at the other end of the landing, in the dark.” Given these circumstances, conforming to typical National Health Service (NHS) care pathways was, therefore, of very little use to these interviewees; they had to make alternative arrangements as it was unlikely that a midwife would arrive in time for the birth.

Nadia was the only interviewee who had previously given birth in a standalone Birth Centre. She spoke very little about her first birth in a hospital, but her next two at the Birth Centre she described as “great,” and “really empowering and wonderful.” She described a room with “beautiful big windows … And it was really magical because the sun would shine, and then it rained, and then it snowed, and it was like all the seasons in one go.” Contrary to what may be presumed, Nadia demonstrates how not all freebirthers are motivated by previous birth trauma or obstetric violence.

Importantly, in this cohort, the women's previous antenatal care was also an important motivator as demonstrated by Fionnuala during her first (non-freebirth) pregnancy. Conforming to the maternity protocol created its own particular psychological dangers. As someone with a history of sexual abuse and eating disorders, she had been very open with the HCPs regarding her background and her subsequent need to give birth in a “safe place,” which for her necessitated a homebirth. Fionnuala recalled

“I was just really open with them [HCPs], thinking that that would help to inform their response to me, but actually, it made the whole situation worse, and I felt-, I felt fat-shamed. I felt bullied. I felt the whole antenatal process was really dehumanising, and that I had to be-, go along with everything they said, in order for them to grant me the permission to have my home birth.”

She continued

“They didn't see me as an individual woman. They just saw me as someone, on paper, that they needed to follow their policies and procedures, with-, rather than actually, tailor my care, in inverted commas, to me.”

What Fionnuala learned from her experiences was that she is not guaranteed individualised care focused on her specific needs. The knowledge she gained from these encounters is that the policies and procedures of the hospital as an institution can take precedence over a woman's psychological needs. The Price of Conformity becomes apparent: if she adheres to the standard maternity protocol, she may be treated as something less than human. This carries its own dangers and, therefore, Fionnuala considered alternative options. Indeed, in her next (freebirth) pregnancy, Fionnuala eschewed all NHS antenatal care.

Other women reported very abusive previous non-freebirth pregnancies. Notably, some of this obstetric violence occurred during previous homebirths, and this has been reported elsewhere (69). However, much of the abuse took place in hospital. The most blatant form of obstetric violence occurred when women were subjected to invasive and intimate procedures with no attempt from the HCPs to ensure informed consent. Such incidents disregarded a woman's right to autonomy and a “voice,” thus silencing her and subjecting her to the will of the person or people around her.

***Cut***

There I am with a spinal block.

I can't feel anything

My legs are up in stirrups.

I didn't know whose legs they were.

I couldn't feel them

I'm like -

I realised they were mine.

I was like

I remember he was Swedish.

“You, when I say ‘Push’ *you have got to push.*”

I'm thinking

I can't feel *anything* now.

I was trying my absolute best.

I did push and *without question*

I was –

Episiotomy.

*Bianca*

Kitty also reported a non-consensual episiotomy (cutting of the perineum). On arrival at a non-UK-based hospital for her first birth, the attending midwife gave Kitty's doula the “heads-up” regarding the name of the obstetrician on call. Kitty described the obstetrician as being “notorious in the community” and nicknamed “The Butcher.”

At some point during her labour and without her knowledge, the HCPs decided that it was time to carry out an episiotomy and assisted birth. Kitty explained

“… [the obstetrician] took my non-verbal cues as consent [to episiotomy], and so the midwife, who was fairly good up until that stage, did perform an episiotomy, and as soon as that happened, the other obstetrician, who I was having the, the problem with, sort of stepped into the situation much closer to me, and just essentially removed [my baby] from my body.”

In this case, the obstetric violence resulted in a “3b tear,” which is “50% through the anus” and “is pretty much, you know, on the verge of as bad as it gets.” It took “two hours” to complete the suturing which was done under general anaesthetic.

In Kitty and Bianca's examples, the women learned the dangers of birthing in an environment where other adults may take control of their birth and their bodies. In Bianca's case, she required additional surgery to correct the poorly sutured episiotomy and for Kitty “the tear was such a big part of my life, it still is, you know, it's the thing that I get the most upset about.” These are pivotal moments in these women's narratives—perhaps even in their lives more generally. As a result, first-hand experience of the potential consequences of medicalised birth triggered these women to seek alternatives and to garner knowledge of birth that is positioned outside of mainstream care.

(c). The Road of Trials

***Rights***

I knew

what I was allowed to do.

I knew

what I could decline.

I knew

I could

basically

just

decline

everything.

*Georgia*

Georgia's I-Poem provides the baseline for women's rights during pregnancy and birth. However, knowing one's own rights is very different to being able to *exercise* those rights. This is the point at which Campbell's (8) and Frank's (9) Road of Trials becomes relevant. In *The Hero with A Thousand Faces*, Campbell (8) references the Road of Trials as part of a more detailed stage of the monomyth entitled “Initiation.” In short, however, the Road of Trials is a “long and really perilous path of initiatory conquests and moments of illumination” (p. 90).

For many of the women in this cohort, the freebirth pregnancy was the period in which they experienced obstacles and resistance. It was also a period of learning, whereby they drew on a range of information sources and created connections with likeminded people. Interviewees continued to better understand the Price of Conformity and the social expectations placed on them as pregnant women and mothers.

It may be presumed that women who freebirth make a firm decision to do so at some point in their pregnancies and then follow a specific course of action. In this cohort, however, the women's decision-making was more fluid. What is striking is that most of the interviewees began their freebirth pregnancies intending to homebirth. This is a crucial point because as the women's narratives and (freebirth) pregnancies progressed, the Price of Conformity became more evident.

Two factors typically contributed to this realisation. The first was when the women wanted to engage with maternity services, secure a homebirth, and exercise their legal rights to decline interventions. This typically emerged as a desire for a “hands-off birth,” which included declining vaginal examinations or frequent foetal heart rate monitoring during labour. Of most note is that it was often the behaviour and responses of NHS maternity staff that made the women seek alternatives and disengage.

Polly described being labelled as “geriatric” and was not supported when she attempted to discuss a homebirth:

“[S]he [midwife] was, like, rolling her eyes and saying that that doesn't happen, and especially not with first-time mothers, and I didn't have a clue what it was like to give birth.”

In Leah's experience, the midwives continually focused on risk when she attempted to arrange a homebirth without monitoring:

“I remember just one appointment they were just listing, like, facts at me about-, and she even said to me, ‘I've been at a birth where the baby’s died,’ you know, ‘And I've experienced loss, and you need to know your-, you need to be sure about this,’ and this was just because I was having, like, this home birth and I wanted it unassisted [“hands-off”] and because I didn't want the monitoring.”

Alicia experienced a similar situation when trying to organise a hands-off homebirth. The resistance she faced included talk of “Death, death in homebirths … the rates of death, mothers dying.”

When Danielle attempted to decline blood tests during her antenatal appointments, she was subjected to continual phone calls from midwives, and during one, she was threatened with social services. She described the experience as “harassment” and it pushed her towards disengaging with maternity staff:

“I just said to him [husband], I said, ‘I don't think I want to have anyone here for the birth. I just want it to be us.’ And he was like, ‘Okay, that’s fine,’ because I think, by that point, he knew that I was … I was really depressed and withdrawn by that point … I was having a-, I don't even know if I was having a difficult … pregnancy, I think it was just very emotional, and I was having a really-, like every day, I was like, ‘Oh, another missed call,’ and it was getting really stressful.”

The second factor was when the women realised they were not receiving the type of care they wished to receive. When the women felt it necessary, they were quick to engage with the hospital for medical support. During her freebirth pregnancy, Cat, for example, reported reduced foetal movements and underwent additional Doppler monitoring. Similarly, Heather had two additional scans and went to the hospital to check reduced movements at 40 weeks. However, if the women felt the appointments were unnecessary, authoritative, or lacking in pastoral care, it triggered thoughts of disengagement. As Jiskra's I-Poem highlights

***Antenatal appointment***

I was like

I felt like

I want some sympathy.

I want someone to just

Smile at me.

Give me some sort of encouragement.

I didn't really get very much of that.

I understand this is really not

I wasn't like kind of expecting it.

I was like

My pregnancy

I really don't-

I don't know how to

I was starting to feel that

What is truly important for me

Is a different type of care than the one I was getting.

*Jiskra*

Eight women specifically referred to staff “box ticking” during NHS antenatal appointments, with Ivy describing it as a “conveyor belt.” An impersonal approach with fractured services meant that the women did not always feel they were being appropriately supported during their pregnancies.

Once women began to consider freebirth and/or disengage with maternity services, the next obstacle was the stigma attached to their decision-making. The interviewees’ recognition of that stigma was apparent in the way that they did not always speak openly about their freebirth plans with the HCPs or the people around them. Leah explained

“I feel like I couldn't speak freely about it, like, even with family or friends, and the medical environment … it’s, like, unheard of really, it’s very frowned upon, and I just felt, you know-, it was very difficult. I had to hide it, you know …”

Mirroring the concerns of other interviewees, Nadia highlighted why she did not tell the HCPs about her plans and disguised her freebirth as a Born Before Arrival (BBA):

“… there was times when I'd say to my husband, shall I just tell them [maternity staff] about-, I just want to have a freebirth like-, and he'd be like, no. Because we'd read about how then sometimes social services would-, people would call them and then there'd be this whole drama, you know? And I thought, no.”

Ophelia highlighted that she needed to “play this carefully” and emphasised what she thought would happen if “people in the profession” found out:

“… you think of the worst-case scenario, they'd take your baby away from you. Like, they would deem me neglectful because I was refusing care and things so I just did not need it.”

Women were aware that HCPs were connected to a wider system that was perceived as somehow authoritative. Without prompting, 10 women voiced their concerns about the potential for NHS midwives to refer them to Children's Services or their local Child Protection Department. Heather described this as “health surveillance” and Ophelia as having “eyes on me”. Consciously silencing herself, Ivy's comment was typical of women's awareness of the situation:

“And I just found that every time I saw her [NHS midwife] I had to bite my tongue because I didn't want, like, to trigger anything, like, her reporting me for things, because it’s tricky waters, isn't it, and first baby.”

Regardless of this stigma, nine women did inform HCPs of their plans. It is clear from the data that the NHS responses were unpredictable and lacked uniformity. Three women did not face any resistance from staff. Other women received different responses. Jiskra received a “dressing down” from her midwife. In Leah's case, she informed an acquaintance of her freebirth plans, and the information filtered back to midwives at the local hospital. She described receiving a telephone call whereby a midwife reported to her that they had “just got through some strange information that you might be having a freebirth.” After Leah confirmed her intention, the midwife stated that she must immediately meet with her:

“… she was, like, ‘I'm coming up to your house now.’ And I was, like, ‘I'm sorry?’ I was, like, ‘What?’ I said, ‘I'm in my (job), I can't come up there,’ and then, honestly, I had to meet them again and go over it all again.”

One important point to emphasise based on the above data during the Road of Trials is the women's difficulty in exercising what Goodwin (70) describes as “the basic ‘natural right’ to be left alone” (p. 107). Although the women were acting lawfully, the HCPs became moral adjudicators of the women's (lawful) decision-making and gatekeepers to the women's own autonomy and bodily integrity. This was particularly apparent when the women struggled to secure “hands-off” homebirths.

(d). Engagement in the Female Network and a Secret Network

Hudson's (5) account of the next stages of the protagonist's journey includes Stage 3: Opportunity to Shine, Stage 4: Dresses the Part, and Stage 5: The Secret World. These stages are too prescriptive to apply to the women's freebirth accounts in a very rigid way. However, when combined, they describe a phase in which the protagonist learns more about herself and her “true nature” (p. 41). She has the opportunity to “shed the clothes that fit other people's expectations of her” (p. 49). Ultimately, the protagonist enters a secret world separate from external authority and which requires some form of “rebellion” (p. 50). She thus moves between the conventions of the Dependent World and the freedom of the Secret World she has created or found. By Stage 6: No Longer Fits Her World, the protagonist “increases her power in the form of self-knowledge, and starts to see her dream as a possible reality” (p. 54).

The stages described by Hudson (5) reflect how the women in this cohort began to move towards freebirth. By reaching out to various people, organisations, and groups and by researching outside of mainstream sources, the interviewees learned of an alternative birthing pathway. Much of this learning was done in secret or drawn from “underground” sources. In essence, the biomedical approach to birth represents the Dependent World, whilst the Secret World, comprising largely of closed online groups and subjugated knowledge sources, reflects the “rebellion” of which Hudson writes.

What becomes immediately clear is that these unofficial sources form part of a Female Network in which women share information with each other and create a knowledge base positioned outside of that most valued by the Dependent World. This has been recognised in literary theory pertaining to the Heroine's Journey as the way in which women create a “matriarchal network … of mother figures, female relatives, friends, mentors, and sisters who build solidarity through teaching maternal history and culture” [(71), p. 5]. Writing about the Heroine's Journey and the way in which women's speech and storytelling have been “belittled and maligned” and branded as merely “gossip,” Tatar (50) argues that such information sharing “knits women together to create networks of social interactions beyond patriarchal control and oversight” (p. 121). In Carriger's (51) understanding of the Heroine's Journey, the protagonist's strength and power derives from her relationship with others and the aid and assistance she sources from those around her (p. x.i.v).

## 9 Storytelling

Primarily, in this cohort, knowledge sharing was centred on the lived experience and the power of storytelling. Freebirthing women tapped into this matrix in various ways, but online sources were by far the most fruitful. The knowledge they obtained was almost exclusively female, often counter-cultural, and existed in spaces where women could typically voice their authentic selves.

The women joined various Facebook groups, not all relating to freebirth. They included, for example, groups for homebirthers, groups for people adopting a natural parenting approach, and groups for women whose pregnancy had gone “post-dates.” One interviewee also mentioned BirthTube and FreebirthTube, in which women go live online whilst giving birth or upload their videos postnatally. The participants also reported watching YouTube videos of birth.

Nine women commented on the benefit of hearing other women's birth stories as freebirth preparation, and six participants sourced many of these stories from the Freebirth Society Podcast. It is worth emphasising the importance of these stories to women and highlighting that the type of knowledge contained in these podcasts is ***not*** the type of information a person could gain from maternity staff. These information sources are in no way reflective of the biomedical approach to birth. Kitty explained

“with my first birth, I actually wanted to become-, my knowledge, I wanted to become like an obstetrician. I wanted to know everything about birth from an obstetrician type thing. So, like, ‘This is the stage of labour,’ and, like, ‘This is how far your cervix will be,’ and all of that was very useless to me actually. And so, on this, this birth [freebirth], I, um, decided to just really listen or read positive stories. So, I went away from reading the, sort of like, manual on how to do it, to just reading stories, and I have to say that that was the best birth preparation I could have asked for.”

Hearing or reading stories told by women about their own births is central to this form of female epistemology. It reflects the power of storytelling and indicates that, in this cohort, the women were frequently searching for knowledge of birth that is removed from the biomedical framework.

Through the online groups, the women made direct contact with other freebirthing women, posing questions and learning from others’ experiences. Using these groups and other networks, some participants arranged face-to-face meetings with women who had previously freebirthed. Five women reported doing this and gaining insight into the lived experiences of other women.

Eight interviewees sourced information from AIMS and Birthrights, national charities run by women—primarily, but not solely—for the benefit of women. Perhaps unsurprisingly, given the charity's support for this research, AIMS played the biggest role in this respect, and the participants spoke of gaining knowledge on their rights and the evidence behind various interventions and procedures.

## 10 Doulas

Doulas played an important part in the Female Network. Doulas are unregulated, non-medical support workers who assist a woman during pregnancy and birth. They do not require formal qualifications to practise, and they are usually paid for on a private basis [(72)]. Doulas and “doulaing” appeared in 13 women's narratives, seven of which related to support during the freebirth pregnancy and birth.

The women's relationships with their doulas were very different to the ones they had with midwives:

***My doula***

Everything was on my terms.

What would you like?

What would you like?

You know

Do you want?

Do you want?

Do you want to just talk

about how you're feeling?

*Ivy*

The ability to interview and select one's own birth support was an important starting point for the type of relationship the women developed with doulas. Bianca explained

“… as it happened, she [doula] had freebirthed her own baby and was really supportive, which was funny because I'd interviewed several doulas and I didn't know why I'd picked this doula and then I, kind of, thought, ‘This is the reason why,’ and I was really happy about it.”

Selecting a doula meant the women could be immediately open about their freebirth plans. A doula who did not share a woman's worldview would not be hired. Ivy's quote demonstrates these power dynamics:

“So, I arranged a meeting with her [doula] and … I said, ‘Look, my plan is to freebirth, so only reply if you'd be comfortable with that, I know a lot of doulas aren't,’ and she replied, and she was like, ‘Oh, I would absolutely love to attend a freebirth.’”

Doulas provided the continuity of carer that was frequently lacking within accounts of NHS maternity care. Interviewees recognised NHS midwives as typically unable to provide continuity of carer for the duration of their pregnancy, birth, and postnatal period. The women who hired doulas recounted calling them for advice and support when they needed it. With the exception of Elsie, the women did not report having personal/direct phone numbers for individual or specific NHS midwives.

Doulas provided emotional support. When meeting her doula for the first time, Jiskra described having a deep conversation about her previous birth “almost like a psychotherapy session.” Similarly, for Fionnuala, her doula provided her with “support” and “was someone to be able to talk it all out with.”

Doulas provided information on women's rights and the law, which is not immediately available within NHS literature. Some women described their doula as liaising with healthcare staff on their behalf. Initially wanting a homebirth, Bianca recalled

“… I wanted them [midwives during labour] to talk to the doula before me. So, basically, I wanted them to sit in the kitchen. And they [midwives] didn't like that. But, because I had the doula and … she had contacts with the, senior people in the midwifery team … so my doula went and spoke to their team, to get that okayed. Which it was.”

In Bianca's example, the doula became the intermediary, negotiating between the interviewee and the maternity team. She used contacts Bianca herself did not have to arrange the type of care Bianca wanted. In effect, the doula became an extension of Bianca's “voice.”

Doulas frequently took on the role of protector. An example of this is evidenced by Jiskra who initially wanted a homebirth and described hiring her doula “to keep the medical professionals away from me.”

In this cohort, when doulas attended a freebirth, they adopted the role of “quietly watching and waiting” while the woman laboured and birthed her baby. An example of a doula carrying out this role during a freebirth is provided by Leah:

“… to be honest, she [doula] didn’t do anything … she was there, she was present, she was holding the space.”

Jiskra described the philosophy of her doula:

“Her whole philosophy is to be non-observing, or, like, being, like, really quiet and, kind of, being in the background, not really engaging with the birthing mother. She was also saying, like, ‘a gaze is not something that’s helpful,’ you know, I think I’ve heard that from other midwives. It’s like they listen rather than—they don’t look, they listen, they don’t touch, they just, kind of, quietly, are a quiet presence that’s removed from the birthing mother. So, she was very much like that.”

As doulas are not medically qualified, for the interviewees who hired them for their freebirth, they could guarantee that their doulas would not examine them or become physically involved in their labour and birth. Their presence facilitated the women's quest to pursue a physiological birth, whereby they were supported, yet not at risk of obstetric violence or unnecessary medicalisation.

## 11 Beyond the Female Network

Beyond the Female Network, the women relied on knowledge that extends beyond that associated with the biomedical model of birth. For four women, religion and spirituality played a role in their freebirth narratives. Fionnuala, for example, held a Blessingway, which is a celebratory ritual attended by women for those entering motherhood. Marion's perspective is outlined in her I-Poem:

***Faith***

I firmly

I, kind of, firmly believe

If I did everything

Within my power

Do all my studying

Got my preparations

Do all my nutrition

If I do everything

In my power

God would take care of the rest.

*Marion*

Nadia was inspired by the account of Mary (Maryam) giving birth to Jesus in the Quran:

“it’s got a very detailed account of her birth and it says, like, how she stood and she … held onto a tree, and used, like, her power, you know, being supported by this tree. And the tree happened to be a date tree, and so dates fell and she ate the dates. And just now, it’s like women are finding out about how fantastic dates are, and how much energy they give and here we had it, like, already, like, eat the dates. And then there was … water under her feet, and I was like, ‘oh my god, that’s a water birth!’ (laughing)”

Similarly, Ophelia read Jackie Mize's *Supernatural Childbirth*, which is based in the Christian faith and, using first-hand testimony and biblical scripture, explores the role of God in conception, pregnancy, and childbirth. For both Marion and Nadia, prayer played a role in their freebirth labours, whilst Ophelia reported the use of prayer as preparation prior to her freebirth. These accounts are examples of women's search for alternative sources of knowledge and support that are not rooted in biomedical discourse.

Practical preparation for the freebirth included decorating the birth space with pictures of birthing women, including, in one case, a slide show of artistic images; practising affirmations, hypnotherapy, meditation, breathing techniques, and yoga; sourcing birth pools, herbs, and aromatherapy oils; praying and being “prayed over” by others; preparing a “sandwich bed” with both old sheets and tarpaulin for the birth, which can be immediately stripped off to reveal fresh blankets to use postnatally; writing birth plans; selecting music play-lists; focussing on nutrition; undertaking first aid research; and learning about cord cutting and lotus births (not cutting the cord and keeping the newborn attached to the placenta until the cord dries and naturally leaves the baby's body). One interviewee also prepared a folder with her husband of all the steps they would take if various medical problems arose during labour. Another worked with two of her friends to prepare them to help her at the freebirth. This included providing them with relevant books and delivering a PowerPoint presentation on what she wanted them to do if various adverse events were to occur.

In total, 13 women spoke of tuning into their own bodies as a source of knowledge in preparation for the birth or during the freebirth itself. The terms the women used included, “listening to my body”, “listen to myself,” “instinctive” feelings, or having a “mind-body connection.” Polly repeatedly referred to a “very strange, sort of, confidence” with regards to her feelings towards giving birth without HCPs. Fionnuala explained her feelings:

“I really took the time to dig deep into my knowledge of myself because I believe that there is no-one more qualified to assess a woman’s level of risk than the mother themselves … I really had faith that I would know if there was something going on in my body, and that actually, by engaging with other people, judging and testing and assessing my body, that actually takes away from my own intuition, and that’s actually not safe.”

This embodied knowledge is an information source that again stands outside of mainstream biomedical discourse. It incorporates both self-reliance and responsibility and requires a woman to actively continue to seek knowledge even during labour and birth.

(e). Apotheosis

Frank (64) interprets Campbell’s (8) term “apotheosis” (p. 127) as the end of the Road of Trials [(9), p. 118]. Within Hudson's (3) schema, this reflects Stage Eleven: Chooses Her Light. It incorporates the protagonist's “transformation and a joyous climax to her story,” where she “is boldly being true to herself in the face of oppressive power” [(3), p. 68–69]. In the accounts of the women in this cohort, this is the stage in which the interviewee—against the values of the Dependent World—ultimately freebirthed her baby.

***Freebirth is…***

For me,

the most incredible thing I've ever done.

I have moments

where I think,

'God, I'm

I'm not having a very good day.

I'm not doing anything

with my life.’

I look back to that experience.

I think,

I gave birth to a baby on my own.

That day after I gave birth,

I really had this strength

that I could do anything

that I wanted to

if I just put my mind to it.

I felt 100% confident

in my skills as a mother.

I just felt

I could do absolutely anything.

I felt like a super-hero.

I wanted to

go out on the street and be just like -

'I just gave birth

to my baby

in my house

on my own

now I can move mountains!’

I always go back to that courage

that I felt

I've said before,

totally,

mind-blowingly incredible.

*Polly*

Detailed accounts of the physiological aspects of women's freebirth experience have been published elsewhere (73), for example, the length of their labours and their approach to birthing their placentas. However, for this paper, it is worth emphasising the emotional aspects the women reported during labour and the euphoria they experienced post freebirth. All the interviewees described positive and happy freebirths with no major issues or medical emergencies. Only Georgia went to the hospital post birth to have medical help with birthing her placenta.

Alicia described the initial stages of her labour:

“I've got a really strong memory of walking up and down the hallway to the kitchen and chopping up fresh mango and pineapple throughout the day … I had the music on low, walking up and down. I felt very confident, I was amazed at how confident I felt. I kept saying the positive birth affirmations that I'd had, and I wish I could remember all of them now, but they were really beautiful. About encouraging the baby to move down, feeling them inside me, and letting your body open. And I remember that I always used to tense up in my previous births, and I, I needed to, so I managed to work out a way-, so I gripped, gripped, my teeth really tight but let the rest of me open. Kept imagining my pelvis opening, but tightening my mouth and my teeth, and that really worked for me, that was perfect …”

Nadia described her initial emotions about her labour:

“I was loving everyone, on my birth ball and happy, and, oh yes, singing and all sorts. Banging the drums.”

Marion reported similar thoughts:

***Happy Labour***

I was happy,

I felt completely at home.

I was so happy.

I was with my friends

and my husband.

I was

I was having a great time.

I was in agony but

I was having a brilliant time.

*Marion*

As the labour progressed, the women did not describe painless births; instead, they reported the coping mechanisms they employed to deal with the intensity of the labour. Georgia stated the following:

“I kind of thought I might be, like, a, quite a calm, quiet birther, but I'm really not, I'm, like, very noisy … And, yeah, just got really noisy. Got in the pool and got [my partner] to, like, top it up with hot water. Um, and I was in the pool for about two hours, probably, just, just that, just, you know, like, resting, moaning, resting, for about two hours …”

Fionnuala described a mixture of pain and pleasure:

“I knew that that was the surge where it was gonna happen (birth), and I had this, like, orgasmic feeling again, like, I mean, I found the labour really-, actually really painful, which I didn't expect to, but that point, it just was, like, I just let out this incredible, like, noise of relief and, like, it just felt-, it actually felt pleasurable for it just to come out, even whilst it really hurt …”

Continuing the idea of the Female Network, it is notable that four women allowed their children—who were all daughters—to witness their freebirths. Fionnuala described her young daughter's response:

“She wasn't fazed at all by me roaring or, um-, she was just, like, so excited … she was having a good look, and yeah, she was just, like, so-, she was just so comfortable in the space. It was-, it was gorgeous.”

Once her baby had been born, she reported her daughter as saying:

“‘Well done, Mama, that’s my mama, that’s my baby.’ And it was just, like, it was just amazing …”

With regards to the women's emotional response to their freebirths, all the women had positive responses. After the birth, Danielle felt “amazing”, and Alicia noted how “it changed me as a mother and a woman.” Elsie commented on how her birthing and freebirthing experiences:

“made me realise how strong I am, yeah, and how, kind of, powerful our bodies are, and how powerful our minds are in controlling what our body does.”

When asked if freebirth had changed her, Ivy answered

“Massively. Absolutely changed me. I think I am so confident now in my own opinion and in knowing exactly what I want and what I don't want …. it’s made me have massive respect for my body, like, huge … Yeah, I think it’s just absolutely a life changing experience, and I think it just made me a better mother, I think. I felt so, like, I just felt, like, on a high, for weeks and weeks and weeks afterwards …”

A final point worth making is that the women took their joy of undisturbed physiological birth and fed their experiences back into the Female Network, thus contributing to this knowledge base. Women became doulas, posted their birth videos online, and spoke informally to other women who queried their birth choices. Notably, the importance of storytelling re-emerges. Kitty's I-Poem provides insight into this:

***My story***

I like to share my story.

I like to help other women.

I just

I think the only mantra

I want people to understand

I don't want

I never want to convince

That’s not my priority.

I never want to tell people it’s, you know,

the safest option and the best option.

I believe those things for myself

For my,

For my situation

I had

My only, sort of, mantra

I want people to understand is that it's a perfectly legitimate option

That’s the only thing I want to add.

That’s why I share my story.

*Kitty*

## 12 Strengths and limitations

The limitations of the study are largely characterised by the difficulties in presenting data from a VCRM study that can be published in typical social science formats. Combining poetry with other outputs that do not reflect a thematic analysis framework creates significant obstacles in the use of the methodology. Of note is the amount of data produced from four readings of an interview transcript and the inability to incorporate much of this into an academic text. Nevertheless, the VCRM does provide a rich and unique way to analyse data, with I-Poetry serving as a potential vehicle for wider public engagement in the form of, for example, creative media such as videos or recitals.

With regards to strengths, freebirthing women are a much-maligned community, and their experiences have only relatively recently been central to social science research. Understanding their experiences as a form of quest and within the context of the Heroine's Journey is new to the literature. Further, not only does the Heroine's Journey provide a workable template to present the data, but its wider use outside of academia provides an opportunity to contextualise broader feminist literature against the experiences of freebirth participants. Potentially, the Heroine's Journey template may also be applicable to other feminist methodologies, or those designed to explore the lived experiences of oppressed groups.

## 13 Conclusion

The Hero’s and the Heroine's Journeys are intrinsic to Western storytelling (8, 51). They provide a template through which experiences can not only be shared and understood but results from VCRM research can be presented. Given that all feminist research presupposes a struggle against patriarchal norms, it is conceivable that the Heroine's Journey template could be applied to a range of feminist methodologies or those that endeavour to platform the experiences of other oppressed or minority groups.

In a society in which pregnancy and childbirth are both pathologised and medicalised (74–77) and ideas of good mothering dictate that women submit to the instruction of HCPs Jenkinson et al. (78, 79), challenging this hegemony by freebirthing is both counter-cultural and difficult. In effect, it becomes a quest of sorts as women must overcome various obstacles to achieve their goal of undisturbed physiological birth. The social forces at play reflect Hudson's (5) Dependent World and represent the social territory through which the interviewees must travel to protect their rights to autonomous birthing and bodily integrity.

This desire to freebirth appeared early in accounts of the women who had precipitous labours and freebirth was a necessity. Yet for other women, the Price of Conformity and the consequences of conforming to typical maternity pathways was a dawning realisation. This realisation was linked to obstetrically violent previous births and their experiences of antenatal care and was combined with their increased knowledge of alternative pathways.

The women understood the maternity system to be authoritative and pregnancy decisions that moved away from expected behaviour as taboo. This led to Secret Worlds forming in which the women tapped into the knowledge of a Female Network. These were largely closed online groups. But they also included hiring doulas, meeting other freebirthers, and conducting research on the lived experience of undisturbed physiological birth via sources such as the Freebirth Society Podcast. Storytelling, in the form of other women's freebirth accounts, was of central importance to the cohort.

Considerable effort was required before the women could achieve what Campbell (8) and Frank (64) describe as “apotheosis” or the climax to the quest narrative. The emotional rewards for this struggle, however, were considerable, with the women describing their experiences as both life-affirming and life-changing. The women then fed the fruits of this experience back into the Female Network, thus serving to inform others.

The Heroine's Journey template is not prescriptive. But its spirit is a useful way of understanding women's freebirth accounts. It reflects the wider understanding feminist authors epitomise in their work, whether that is in Kate Chopin's *The Awakening* or Margaret Atwood's *Handmaid's Tale.* As the VCRM methodology attempts to demonstrate and subvert, women battle to be heard in a patriarchal society, and this is an issue that continues to manifest in various guises. The struggle for women to give birth in a way that reflects their own worldview and retains their autonomy and bodily integrity is very real. Presenting The Freebirth Study as both a quest and a Heroine's Journey is emblematic of that struggle.

## Data Availability

The datasets presented in this article are not readily available because given the small population of freebirthers, participants may become identifiable. Requests to access the datasets cannot be made, further enquiries can be made to the corresponding author.
